# Comprehensive analysis of LncRNAs expression profiles in an in vitro model of steatosis treated with Exendin-4

**DOI:** 10.1186/s12967-021-02885-4

**Published:** 2021-06-02

**Authors:** Khaoula Errafii, Neyla S. Al-Akl, Olfa Khalifa, Abdelilah Arredouani

**Affiliations:** 1 College of Health and Life Sciences, Hamad Bin Khalifa University, Qatar Foundation, Doha, Qatar; 2 Diabetes Research Center, Qatar Biomedical Research Institute, Hamad Bin Khalifa University, Qatar Foundation, PO Box: 34110, Doha, Qatar

**Keywords:** Steatosis, NAFLD, Exendin-4, LncRNAs, HepG2, GLP-1R agonist

## Abstract

**Background and aims:**

The hallmark of non-alcoholic fatty liver disease (NAFLD) is the excessive hepatic lipid accumulation. Currently, no pharmacotherapy exists for NAFLD. However, the glucagon-like peptide-1 receptor agonists have recently emerged as potential therapeutics. Here, we sought to identify the long non-coding RNAs (LncRNAs) associated with the steatosis improvement induced by the GLP-1R agonist Exendin-4 (Ex-4) in vitro.

**Methods:**

Steatosis was induced in HepG2 cells with oleic acid. The transcriptomic profiling was performed using total RNA extracted from untreated, steatotic, and Ex-4-treated steatotic cells. We validated a subset of differentially expressed LncRNAs with qRT-PCR and identified the most significantly enriched cellular functions associated with the relevant LncRNAs.

**Results:**

We confirm that Ex-4 improves steatosis in HepG2 cells. We found 379 and 180 differentially expressed LncRNAs between untreated and steatotic cells and between steatotic and Ex-4-treated steatotic cells, respectively. Interestingly, 22 upregulated LncRNAs in steatotic cells became downregulated with Ex-4 exposure, while 50 downregulated LncRNAs in steatotic cells became upregulated in the presence of Ex-4. Although some LncRNAs, such as MALAT1, H19, and NEAT1, were previously associated with NAFLD, the association of others with steatosis and the positive effect of Ex-4 is being reported for the first time. Functional enrichment analysis identified many critical pathways, including fatty acid and pyruvate metabolism, and insulin, PPAR, Wnt, TGF-β, mTOR, VEGF, NOD-like, and Toll-like receptors signaling pathways.

**Conclusion:**

Our results suggest that LncRNAs may play essential roles in the mechanisms underlying steatosis improvement in response to GLP-1R agonists and warrant further functional studies.

**Supplementary Information:**

The online version contains supplementary material available at 10.1186/s12967-021-02885-4.

## Introduction

Non-alcoholic Fatty Liver Diseases or (NAFLD) is a broad term that covers the whole spectrum of fatty liver disease, including steatosis, steatohepatitis, fibrosis, and cirrhosis [1]. NAFLD's hallmark is the excessive cytoplasmic lipid accumulation in liver cells not attributed to alcohol consumption, viral infections, or medication [2]. The NAFLD's pathogenesis and pathophysiology involve intricate interactions between genetic predisposition and environmental risk factors such as obesity, insulin resistance, diabetes mellitus, and dyslipidemia [3]. NAFLD's global prevalence is about 25%, but it attains 30% in some regions like the Middle East, South America, and South Asia [4]. Given the relentless rise in obesity rates globally, it is estimated that NAFLD incidence will keep increasing, especially in the absence of effective treatment. As of today, no approved pharmacotherapy exists for NAFLD [5]. Weight loss based on diet and physical activity is the only intervention proven to improve liver function effectively, reduce NAFLD's severity, and positively impact glycemic control and vascular function [6]. However, weight loss is notoriously difficult to achieve and even more challenging to maintain.

In the recent few years, Glucagon-like peptide-1 receptor agonists (GLP-1RAs), already approved for the treatment of type 2 diabetes (T2D) [7], have emerged as potential drugs for the treatment of NAFLD due to their appetite and food intake reducing effects [8], and their ability to increase lipid oxidation [9]. GLP-1 is a multifunctional hormone secreted by the intestine L cells [10]. Among other functions, GLP-1 regulates glycemia via the stimulation of glucose-dependent insulin release and the decrease of glucagon secretion [11], promotes pancreatic β-cell proliferation, and reduces satiety and food intake via actions on centers in the central nervous system [12]. Some GLP-1RAs, like liraglutide and dulaglutide [13], are already used to manage T2D and obesity in humans [14], as they can mimic the effects of GLP-1 in vivo. Recent in vivo and in vitro studies have tested the impact of the GLP-1RAs on liver fat content and have shown promising results [8]. Therefore, these agents were suggested as potential options for managing and slowing NAFLD progression [15].

Emerging evidence indicates that GLP-1RAs can markedly reduce hepatic steatosis in vitro by modulating the expression of genes involved in lipid metabolism (inhibiting fatty acid synthesis-related genes and enhancing the expression of fatty acid oxidation-related genes) [16]. However, little is known about the role of long non-coding RNAs (LncRNAs) in the reported GLP-1RA-induced hepatic steatosis improvement.

LncRNAs represent a diverse class of transcribed RNA molecules with a length of more than 200 nucleotides that do not encode proteins [17]. LncRNAs have roles in many liver functions, particularly in lipid metabolism, inflammation, cell apoptosis, and development [3]. Several previous studies have investigated the dysregulation and modulation of LncRNAs expression in NAFLD [3, 18], and a myriad of LncRNAs, including MALAT1, NEAT1, H19 and CCAT1 were linked to different stages of NAFLD [19–22].

In the present study, we used HepG2 cells treated with oleic acid (OA) as an in vitro model for steatosis to investigate the potential involvement of LncRNAs in the protective effect of the GLP1RA Ex-4 on hepatic steatosis.

## Materials and methods

### Cell culture and reagents

The HepG2 cell line (ATCC HB-8065) was cultured in a humidified atmosphere at 5% CO_2_ and 37 °C in a DMEM medium (31966047, Gibco, Massachusetts, USA) containing 10% fetal bovine serum (FBS) (10500064, Gibco, Massachusetts, USA) and 100 mg/l penicillin and streptomycin (15070063, Gibco, Massachusetts, USA). All the experiments were performed with cells passaged less than 25 times. The OA solution was prepared, as previously reported [23]. Briefly, the powder OA (O-1008, Sigma-Aldrich, Germany) was dissolved by sonification at a final concentration of 12 mM in PBS that contained 11% fatty acid-free bovine serum albumin (BSA; 0215240110, MP Biomedicals, Santa Ana, CA, USA). The solution was shaken overnight at 37 °C, filtered with a 0.22 µm filter, and stored in aliquots at 4 °C. Exendin-4 (Ex-4) was purchased from Tocris (E7144-0.1MG, Tocris, Minneapolis, Minnesota), aliquoted, and stored at − 20 °C. Fresh OA and Ex-4 aliquots were used for each experiment.

### Induction of steatosis with oleic acid and treatment with Ex-4

HepG2 cells were seeded at a density of 4 × 10^5^ cells/well in 6-well plates until 70% confluence was reached. They were then starved for 6 h in DMEM containing 1% fatty-acid free bovine serum albumin (FFA-BSA) instead of 10% fetal bovine serum. Upon starvation, steatosis was induced by treating the cells with 400 μM OA for 16 h in DMEM medium containing 10% BSA. Cells were then treated for 3 h with a fresh DMEM solution containing 400 µM OA in the presence or absence of 200 nM Ex-4.

### BODIPY 493/503 staining of lipid droplets

HepG2 cells were grown in glass-bottom dishes and treated as mentioned above. They were then incubated for 10 min in the dark with 0.2 μM boron-dipyrromethene (BODIPY) 493/503 (D3922, Thermo Fisher Scientific, MA, USA), which labels specifically intracellular neutral lipids, and 1 min with 1 μM DAPI (10236276001 Roche, Switzerland) to stain the nuclei. Imaging of the cells was performed on a Zeiss LSM 870 confocal microscope. The channels' exposure times were independently set to maximize the signal while minimizing the number of cells with expression levels above saturation to optimize the assay's dynamic range. Once set for each channel, all images in that channel were collected at the same exposure. We used ImageJ software (version 1.8.0, NIH, USA) to analyze the images. The intracellular lipid accumulation was quantified by dividing the BODIPY fluorescence intensity over that of the DAPI. For each treatment condition (untreated, steatotic, and Ex-4-treated steatotic cells), two independent researchers analyzed 200 individual cells from three different experiments.

### RNA extraction

We extracted total RNA from cells under different treatment conditions using the Pure Link RNA Mini kit (12183025, Invitrogen, USA), according to the manufacturer's instructions. The RNA samples were immediately frozen at − 80 °C until use. Before library preparation, we used an RNA broad range assay kit (Q10211, Invitrogen, Carlsbad, CA, USA) and Qubit 2.0 (Thermo Fisher Scientific, USA) to measure the RNA concentration. The RNA quality was assessed using the Agilent RNA 6000 Nano Kit (5067-1511, Agilent, CA, USA) and Agilent 2100 Bioanalyzer (Agilent Technologies) as per the manufacturer's instructions.

### Library preparation and RNA sequencing

We used a starting input material of 100 ng of RNA for the library preparation using TruSeq RNA Access Library preparation kit (RS-301-2001 and RS-301-2002, Illumina, San Diego, CA, USA) as per the manufacturer's instructions. Briefly, the RNA was fragmented into small pieces under high temperature using divalent cations. The RNA fragments were immediately reverse transcribed to first-strand cDNA using random hexamers. Following the first strand, the second strand was synthesized by incorporating dUTP instead of dTTP. The sequencing adaptors were ligated to the double-stranded cDNA followed by a single "A" nucleotide adenylation at the 3' end of blunt fragments. The final library was created by capturing the regions of the transcriptome using sequence-specific probes. The yield of cDNA libraries was quantified using the Qubit dsDNA HS assay kit (Q32855, Invitrogen), and the size distribution of the cDNA libraries was determined using the Agilent 2100 Bioanalyzer DNA1000 chip (Agilent Technologies). The clusters were generated on a cBot cluster generation system (Illumina), and sequencing was done on Hiseq 4000 with 150 bp paired-ends.

### Functional annotation analyses

Paired de-multiplexed fastq files were generated using the Linux command line, and initial quality control was performed using FastQC. Paired fastq files were uploaded and aligned to the hg19 human reference genome in CLC Genomics Workbench-12 (Qiagen) using default settings. The abundance of transcripts was measured as the score of TPM (transcripts per million) mapped reads in CLC Genomics Workbench 12. Abundance data were subsequently subjected to differential gene expression using built-in statistical analyses recommended in CLC Genomics protocol with 2.0-fold change and P value cutoff < 0.05.

To gain insight into the role of LncRNAs in the observed Ex-4-induced steatosis improvement, we used the LncPath package in R (https://cran.r-project.org/web/packages/LncPath/) to identify functional pathways influenced by the combinatorial effects of the LncRNAs of interest based on a global network propagation algorithm [24]. Briefly, the LncRNAs are first uploaded to R studio. A coding non-coding network (CNC) is then constructed based on a LncRNA-mRNA interaction network. The LncRNAs are then mapped into the CNC network. The program was instructed to use the Kyoto Encyclopedia of Genes and Genomes (KEGG) pathway database (https://www.genome.jp/kegg/pathway.html) to identify relevant biological functions and pathways.

### Quantitative reverse transcription PCR (qRT-PCR)

We used the High-Capacity cDNA Reverse Transcription kit (4368813, Applied Biosystems, Foster City, CA, USA) to reverse-transcribe 1 µg of RNA into cDNA. Up-regulation and down-regulation of a set of transcripts were validated using qRT-PCR on the QuantStudio 12 Flex qPCR (Applied Biosystems USA). Real-time PCR was performed using PowerUp™ SYBR™ Green Master Mix (A25780, Applied Biosystems, USA), and relative levels of transcripts were determined from their respective CT values normalized against β-actin transcript levels. The validated LncRNAs and the respective sequences of primers used for PCR are listed in Table 1.

**Table 1:** Primer sequences for qRT-PCR. The primers used for MALAT1, NEAT1, and H19 were previously published, while the primers for TP73-AS1, ABALON, and HOXA10-AS were designed using primers3 (http://www.ncbi.nlm.nih.gov/tools/primer-blast/)

Primer	Sequence
MALAT1 [25]	F:GAATTGCGTCATTTAAAGCCTAGTT
	R:GTTTCATCCTACCACTCCCAATTAAT
NEAT1[26]	F: GTGGCTGTTGGAGTCGGTAT
	R: ACCACGGTCCATGAAGCATT
TP73-AS1	F:CCGGTTTTCCAGTTCTTGCAC
	R:GCCTCACAGGGAAACTTCATGC
ABALON	F:CCCCCTCCAGGTACCAGAAC
	R:CCACTGGTGCTTTCGATTTGA
H19 [27]	F:TCAGCTCTGGGATGATGTGGT
	R:CTCAGGAATCGGCTCTGGAAG
HOXA10-AS	F: CCCAGTAAGCCAAAGTCAAGCC
	R: CTGAGGTCAATGGTGCAAAGG

### Statistical analyses

We performed all statistical analysis and graphing using GraphPad Prism 9.0 software (GraphPad Prism v9, La Jolla, CA, USA). We used unpaired one-way analysis of variance (ANOVA) to evaluate the significance of differences between the mean values of different experimental groups. Multiple comparisons between experimental groups were adjusted with the Bonferroni correction. For qRT-PCR experiment, we used a t-test for independent samples to compare the expression level between different groups. A *p* value < 0.05 was considered statistically significant unless otherwise indicated.

## Results

### Exendin-4 reduces lipid accumulation in HepG2

**Fig. 1:**
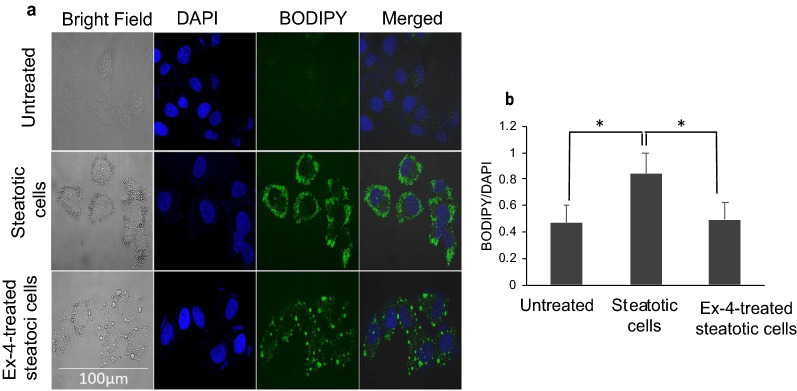
Exendin-4 reduces Oleic acid-induced lipid accumulation in HepG2 cells. In all experiments, HepG2 cells were starved for 6 h and then treated with 400 μM Oleic acid for 16 h. Afterwards, cells were treated for 3 h with a fresh DMEM solution containing 400 µM OA in the presence or absence of 200 nM Ex-4. **a** Confocal imaging of lipid droplets after staining with 0.2 mM BODIPY 493/503 (green) and DAPI (blue). The white dots in the bright field images indicate lipid droplets. **b** Quantification of the lipid content with the BODIPY/DAPI fluorescence ratio in steatotic cells relative to untreated cells, and in Ex-4-treated steatotic cells relative to steatotic cells. For each treatment condition (untreated, steatotic, and Ex-4-treated steatotic cells), two independent researchers analyzed 200 individual cells from three separate experiments. Values are expressed as the mean ± SE (n = 3). *p < 0.05

We determined OA's optimal concentration required to induce steatosis by treating HepG2 cells with increasing OA concentrations (0, 200 µM, 300 µM, 400 µM, and 500 µM) overnight and by quantifying triglycerides (TGs) accumulation (data not shown). A significant accumulation of TGs was obtained with 200 µM OA, but the saturating levels of TGs were achieved with 400 µM (p < 0.001, relative to untreated). Therefore, we used 400 µM OA to induce steatosis in all our experiments. Moreover, we compared TGs content between untreated cells, steatotic cells, and Ex-4 -treated steatotic cells. There were statistically significant differences between group means as determined by one-way ANOVA (F(2, 6) = 6.4, p = 0.032). Post hoc analysis correcting for multiple comparisons revealed significant differences between untreated cells and steatotic cells (p < 0.05), and between steatotic cells and Ex-4-treated steatotic cells (p < 0.05) (Fig. 1). This observation suggests that Ex-4 reduces OA-induced lipid accumulation.

### Identification of differentially expressed LncRNAs (DELs)

**Fig. 2:**
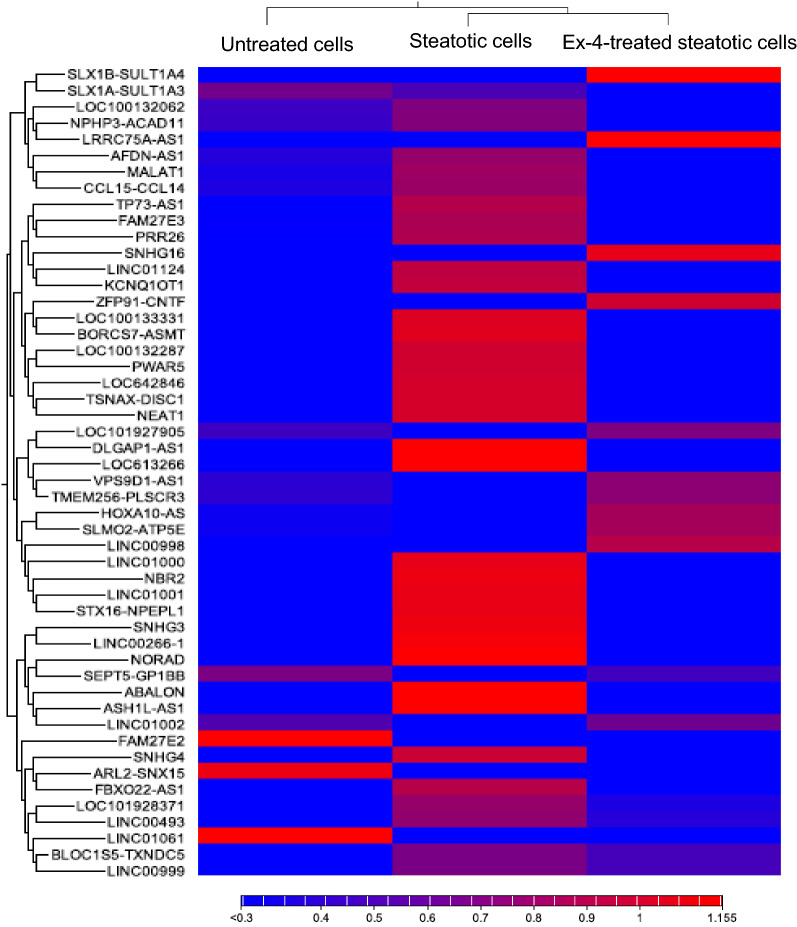
Hierarchical Clustered Heatmaps of the top 50 LncRNAs showing differential expression between untreated, steatotic, and Ex-4-treated steatotic cells. Each row represents one LncRNA, and each column represents one sample. The expression level of each transcript in a single sample is depicted according to the color scale. Red and blue indicate up-regulation and down-regulation, respectively. The experiment was performed in triplicate

To investigate the role of LncRNAs in the observed Ex-4-induced improvement in steatosis, we sequenced the total RNA extracted from untreated, steatotic, and Ex-4-treated steatotic cells in triplicates using a Hiseq 4000 platform. After removing the low-quality reads and adaptors, untreated, steatotic, and Ex-4-treated steatotic cells, respectively showed, 25396454, 36665640, and 32101936 read depths. Sample log folds hierarchical clustering based on differentially expressed transcripts revealed distinct clustering of LncRNAs between the three treatment conditions. Figure 2 displays the differential expression of the top 50 LncRNAs between the three groups. A full list of all the DELs between the three groups is shown in Additional file 1: Table S1.

**Fig. 3:**
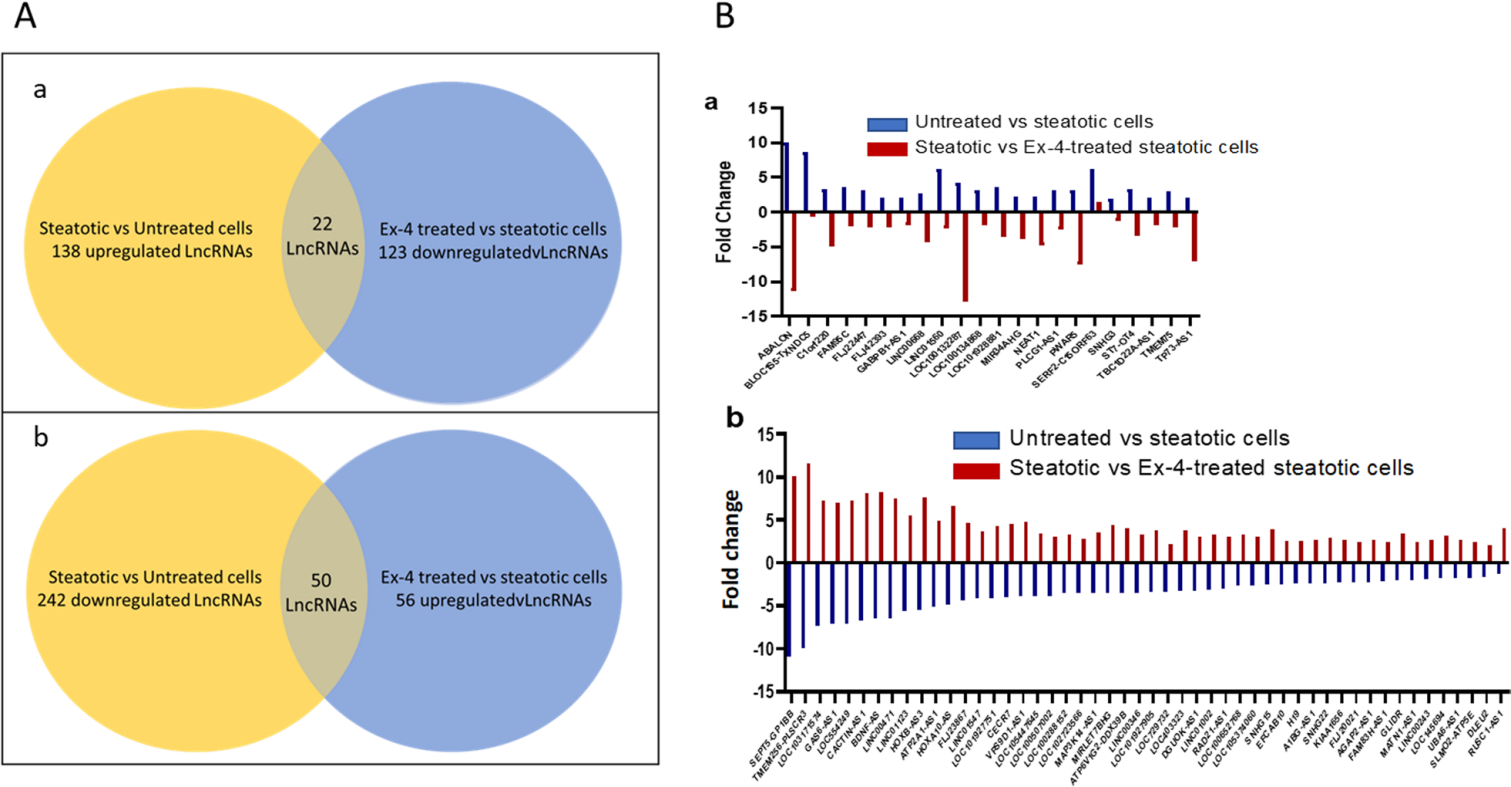
LncRNA transcriptional portrait between untreated, steatotic, and Ex-4-treated steatotic cells. **A** Venn Diagram of differentially expressed LncRNAs. The 22 LncRNAs at the intersection are upregulated in steatotic cells and downregulated in Ex-4 treated steatotic cells (**a**). The 50 LncRNAs at the intersection are downregulated in steatotic cells and upregulated in Ex-4 treated steatotic cells (**b**). **B** Fold change of 22 LncRNAs upregulated in steatotic HepG2 cells and downregulated in Ex-4-treated steatotic Cells (**a**) and fold change of 50 LncRNAs downregulated in steatotic HepG2 cells and upregulated in Ex-4-treated steatotic Cells (**b**). The experiment was performed in triplicate

We identified 379 significant DELs, with 138 upregulated and 241 downregulated in steatotic, compared to untreated cells (Fig. 3A(a) and Additional file 1: Table S1). These 379 DELs may be associated with the lipid accumulation in HepG2 cells in response to OA treatment. On the other hand, 180 significant DELs, with 58 upregulated and 122 downregulated, were identified in Ex-4 treated steatotic cells compared to steatotic cells (Fig. 3A(b) and Additional file 2: Table S2). These 180 DELs may be associated with the positive effect of Ex-4 on steatosis. Interestingly, 22 LncRNAs upregulated in steatotic cells were downregulated in the presence of Ex-4 (Fig. 3A(a) and B(a), and Additional file 3: Table S3), whereas 50 LncRNAs downregulated in steatotic cells were upregulated by in Ex-4-treated steatotic cells (Fig. 3A(b) and 3B(b), and Additional file 4: Table S4). These 72 DELs may be functionally related with, and maybe more critical to, the protective effects of Ex-4 on OA-induced steatosis.

### Validation of differentially expressed LncRNAs by qRT-PCR

**Fig. 4:**
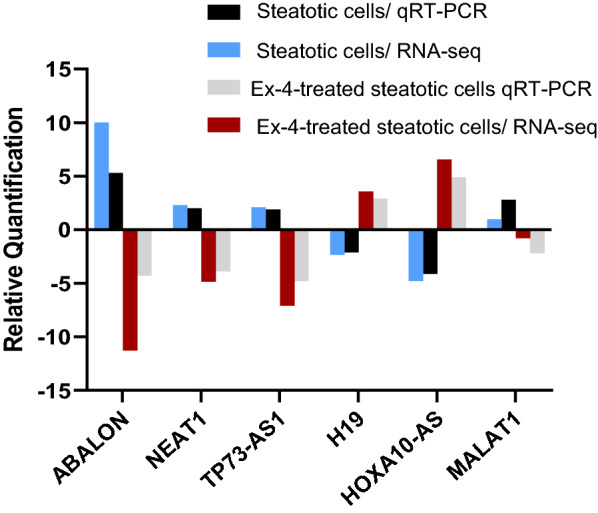
Validation of the differential expression of a set of LncRNAs with qRT-PCR. Comparison of the expression of LncRNAs ABALON, NEAT1, TP73-AS1, H19, MALAT1, and HOXA10-AS between steatotic cells and Ex-4-treated steatotic cells using qRT-PCR and RNA sequencing. The primers used for each LncRNA are shown in Table 1. The experiment was performed in triplicate

To validate the differential expression results in RNA-seq data, we performed qRT-PCR analysis on a set of LncRNAs (Fig. 4). The qRT-PCR results were consistent with the RNA-seq data and revealed differentially expressed LncRNAs between Steatotic cells and Ex-4-treated steatotic cells.

### Functional enrichment analysis

**Fig. 5:**
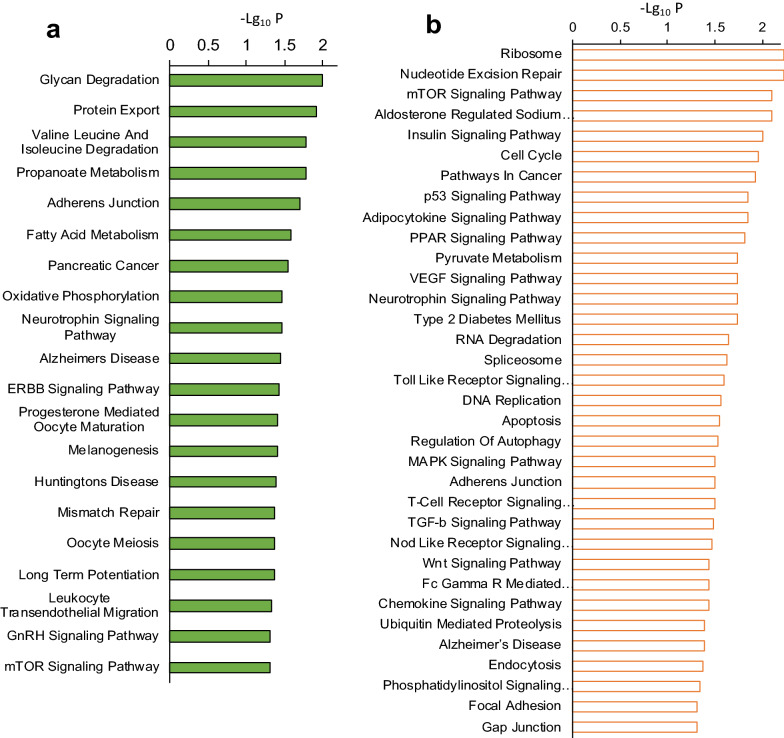
Functional enrichment analysis of differentially expressed LncRNAs. **a** Signalling pathways and biological processes associated with the 22 LncRNAs that are upregulated in steatotic cells and downregulated in Ex-4-treated steatotic cells. **b** Signalling pathways and biological processes associated with the 50 LncRNAs that are downregulated in in steatotic cells and upregulated in Ex-4-treated steatotic cells. The experiment was performed in triplicate

In order to identify the biological processes that might be affected by the sets of 22 and 50 LncRNAs, we uploaded the two sets separately to the LncPtah package in R. The software was instructed to use the Kyoto Encyclopedia of Genes and Genomes (KEGG) pathway database (https://www.genome.jp/kegg/pathway.html) to identify the relevant functional pathways. The functional enrichment analysis results revealed that the 22 LncRNAs that were upregulated in steatosis and downregulated upon Ex-4 treatment were associated with several critical biological and molecular processes such as glycan degradation, protein export, fatty acid metabolism, GnRH, and mTOR signalling pathways (Fig. 5). On the other hand, the analysis revealed that the 50 LncRNAs that were downregulated in steatosis and upregulated upon Ex-4 exposure were significantly associated with essential processes and molecular pathways, including insulin, PPAR, Wnt, mTOR, p53, TGF-β, VEGF, NOD-like, and Toll-like receptors signaling pathways as well as pyruvate metabolism and type 2 diabetes (Fig. 5).

## Discussion

We have investigated the potential implication of LncRNAs in the Ex-4-induced improvement of steatosis in HepG2 cells. We detected significant changes in the expression of several LncRNAs between untreated and steatotic cells and between steatotic cells and steatotic cells treated with Ex-4. The differentially expressed LncRNAs are involved in various critical biological processes and signaling pathways directly or indirectly relevant to NAFLD.

Because of the concomitant skyrocketing rates of obesity and insulin resistance worldwide, NAFLD has become a global health problem [28]. To date, no approved pharmacotherapy exists for NAFLD. However, recent human and animal studies have documented a potential beneficial effect of the GLP-1R agonists on the disease [8, 29]. The mechanisms that underlie this positive effect are yet to be elucidated.

Although thousands of LncRNAs have been identified in recent years, the possible role of LncRNAs in the positive effect of GLP-1R agonism on NAFLD has never been researched yet. In this study, we performed untargeted profiling of LncRNAs to elucidate the molecular mechanisms underlying the observed decrease in OA-induced lipid accumulation in response to Ex-4 treatment in an in vitro steatosis model. We opted for an in vitro model of steatosis to overcome the pleiotropic effects that characterize the action of GLP-1R agonists in vivo [12]. Indeed, among other effects, GLP-1R agonists promote weight loss, induce satiety, and reduce insulin resistance, effects that can all improve NAFLD independently of direct activation of hepatic GLP-1Rs [30].

Interestingly, some studies have reported the lack of GLP-1R expression in hepatic cells [31], leading to the hypothesis that the effect of GLP-1R agonists on NAFLD is not mediated by direct GLP-1R activation. However, previous studies have documented the expression of GLP-1R by human hepatocytes [32, 33]. In our hands too, the HepG2 cells express GLP-1R (data not shown). Although LncRNAs usually do not translate into proteins, they regulate protein-coding genes and related signaling pathways involved in multiple diseases [34], including NAFLD [3]. Our data shows that there were differences in LncRNA expression profiles between untreated and steatotic HepG2 cells. Compared to untreated cells, 138 and 242 LncRNAs were found to be respectively upregulated and downregulated after steatosis induction with OA. On the other hand, we found 56 and 123 LncRNAs that were respectively upregulated and downregulated in Ex-4-treated steatotic cells relative to steatotic cells. The function of several of the differentially expressed LncRNAs in our study is unknown, while many others are known to be associated with different diseases such as cancer [35], and a set of them was previously associated with NAFLD [3].

Interestingly, 22 and 50 LncRNAs were respectively upregulated and downregulated in steatotic compared to untreated cells, but the exposure to Ex-4 reversed the direction of expression of these LncRNAs. This reversal of expression suggests that those 72 LncRNAs might be crucial for the significant reduction in lipid accumulation induced by Ex-4.

One of the 22 LncRNAs is NEAT1, which promotes hepatic lipid accumulation via the regulation of miR-146a-5p/ROCK1 in NAFLD [36]. Notably, the down-regulation of NEAT1 was suggested to alleviate the NAFLD via the mTOR/S6K1 signaling pathway [37]. Interestingly, mTOR signalling pathway is one of the important pathways we identified in our functional analysis. It was also recently suggested that NEAT1 could regulate fibrosis, inflammatory response, and lipid metabolism via the miR-506/GLI3 axis [22]. Furthermore, NEAT1 could promote steatosis via enhancement of estrogen receptor-mediated AQP7 expression in HepG2 cells [38]. Together with our findings, these studies indicate that NEAT1 might play an essential role in alleviating lipid accumulation in response to Ex-4 treatment.

The expression of the LncRNA MALAT1 was also significantly upregulated by OA induced steatosis and downregulated by treatment with Ex-4. The association of MALAT1 to NAFLD is documented in several studies. Thus, MALAT1 is suggested to promote hepatic steatosis and insulin resistance by enhancing triacylglycerol biosynthesis through the increase of nuclear SREBP-1c protein stability [20]. Moreover, the expression of MALAT1 is dose-dependently increased in HepG2 cells and primary mouse hepatocytes exposed to different doses of palmitate. Knockdown of MALAT1 significantly reduces the palmitate-induced TG accumulation [20]. MALAT1 is also suggested as a common molecular driver in NASH's pathogenesis and chronic immune-mediated liver damage [39]. The Ex-4-induced down-regulation of MALAT1 in our study may, therefore, play s a critical role in the steatosis improvement we observe upon Ex-4 treatment.

ABALON is another LncRNA whose expression is upregulated in steatotic cells and downregulated in Ex-4-treated steatotic cells. This lncRNA is known to be upregulated in cancers [40]. To date, no known role of this LncRNA in NAFLD has been reported, and further investigations are warranted to understand better its role in steatosis and in the effect of Ex-4 we observed.

The lncRNA TP73-AS1 plays a crucial role in many different carcinomas, including hepatocellular carcinoma [41]. TP73-AS1 regulates proliferation, invasion, migration, apoptosis, and in vivo and in vitro chemoresistance cancer mechanisms through different signaling pathways. No known role of this LncRNA has been reported in other diseases, and more investigations are needed to understand better the differential expression we see in our steatosis model.

The LncRNA H19 is one of the first LncRNAs discovered and associated with liver disease [42]. In our hands, its expression is decreased in the steatotic cells and increased following Ex-4 treatment. However, previous studies have reported overexpression of H19 in primary hepatocytes from a NAFLD mouse model and in steatotic HepG2 and Huh-7 cell lines [43]. We have no explanation for this discrepancy. However, it is worth mentioning the differences between our protocol and the others. For example, Wang and colleagues [43] observed an up-regulation of H19 in primary hepatocytes after 72 h treatment with OA (0.5 mM), compared to 16 h (400 µM) in our study. Besides, in the mouse model of NAFLD they used, the significant H19 up-regulation was only observed after 16 weeks of high-fat diet regimen, while in Liu's study [21], the H19 up-regulation was detected after 8 weeks of high-fat feeding. Additionally, Liu et al., [21] treated the HepG2 and Huh-7 cells with a mixture of 1 mM FFAs, containing oleic and palmitic acids, for 24 h to induce steatosis, as compared to 16 h (400 µM) in our study. The fact that we were able to reproduce the expression changes previously reported for the LncRNAs NEAT1 and MALAT1 gives us confidence in our protocol and analysis. However, further experiments are required to elucidate the above-said discrepancy fully.

The lncRNA HOXA10-AS is downregulated by OA treatment and upregulated after Ex-4 exposure. HOXA10-AS is a LncRNA that promotes cell growth and survival by activating HOXA10 gene expression in glioma [44] and its silencing decreases proliferation [45]. To date, no data that directly or indirectly associates HOXA10-AS with NAFLD are available. Because of the significant changes in expression we observed, this LncRNA deserves further investigation into its role in steatosis developments and improvement with Ex-4.

A relevant finding in our study was the important biological processes and signaling pathways identified by the functional annotation analysis using the sets of DELs. Several of the cellular functions such as lipid metabolism, pyruvate metabolism and type 2 diabetes are directly relevant to NAFLD [46–48]. Furthermore, a number of signaling pathways previously implicated in different stages of NAFLD were also identified, including the signaling pathways of Wnt [38], insulin [49]; mTOR [26], TGF-β [50], NOD [51], TOLL-like receptor [52], VEGF [53], and PPARs [54].

Interestingly, some of the signaling pathways above were associated with the effect of the GLP-1R agonist liraglutide on NAFLD [55]. Further studies are warranted to unravel how all these signaling pathways' combined effect leads to improvement of steatosis upon treatment with GLP-1R agonists.

We acknowledge the limitations of using the HepG2 as an in vitro steatosis model to investigate an important question such as the mechanisms involved in the protective effect of the GLP-1R agonists. Several cell types from multiple tissue sources that involve complex hormone interactions are implicated in lipid metabolism; thus, the effects of Ex-4 we observed at the single-cell level may differ when applied to the whole organism. While the role of LncRNAs in the protective effect of GLP-1R agonists in steatosis has not been researched in vivo, there is ample evidence that these drugs do reduce fat liver content both in NAFLD patients and animal models.

Furthermore, in testing the effect of Ex-4 on the OA-induced steatosis, we used the absence of Ex-4 as the control, which we believed is a good and sufficient control, instead of using another peptide that is analogous or of the same length as Ex-4 for example. This would have been useful to distinguish between biochemical actions and physical influences of the EX-4 structure.

Our findings in HepG2 are a first step towards further investigations in vivo to deeply characterize the role of LncRNAs in the positive effect of GLP-1R agonists on NAFLD. Furthermore, we have used 400 µM of OA to establish our steatosis cell model based on a dose–response experiment and is similar to what has been used in other studies. This concentration is within the physiological range in healthy individuals. Indeed, Abdelmagid et al., [56] have determined the average concentrations of a set of 61 fatty acids, including oleic acid, in plasma total lipids from an ethnically diverse population of healthy young Canadian males and females (Total n = 826) and found that the concentration of oleic acid ranges between 179 µM and 3210.5 µM with a mean value at 1285.5 ± 417 µM. However, the majority of the individuals tested had oleic acid concentration between 300 and 450 µM. Therefore, the concentration of OA we used within the expected physiological range of healthy individuals.

## Conclusion

Our results indicate that the GLP-1R agonist Ex-4 reduces lipid accumulation potentially through the modulation of the expression of LncRNAs that target several genes involved in a myriad of signaling pathways, including lipid metabolism, insulin resistance, and others. Our findings may open a new avenue toward a better understanding of the molecular mechanisms associated with NAFLD's pathogenesis and provide potential novel biomarkers or candidate drug targets for NAFLD. A thorough and comprehensive in vivo investigation of the identified differentially expressed LncRNAs, and their target genes is warranted in the future.

## Supplementary Information

**Additional file 1: Table S1.** DELs (379) of between steatitic and untreated cells.

**Additional file 2: TableS2.** DELs (180) between steatitic and Exendin-4-treated steatotic cells.

**Additional file 3: Table S3.** lncRNAs (22) upregulated in steatotic cells and downregulated in Exendin-4-treated steatotic cells.

**Additional file 4: Table S4.** lncRNAs (50) downregulated in steatotic cells and upregulated in Exendin-4-treated steatotic cells.

## Data Availability

The datasets used and/or analyzed during the current study are available from the corresponding author on reasonable request.

## References

[1] Vanni E, Bugianesi E, Kotronen A, De Minicis S, Yki-Järvinen H, Svegliati-Baroni G (2010). From the metabolic syndrome to NAFLD or vice versa?. Dig Liver Dis.

[2] Pei K, Gui T, Kan D, Feng H, Jin Y, Yang Y, Zhang Q, Du Z, Gai Z, Wu J, Li Y (2020). An overview of lipid metabolism and nonalcoholic fatty liver disease. Biomed Res Int.

[3] Khalifa O, Errafii K, Al-Akl NS, Arredouani A (2020). Noncoding RNAs in nonalcoholic fatty liver disease: potential diagnosis and prognosis biomarkers. Dis Markers.

[4] Mitra S, De A, Chowdhury A (2020). Epidemiology of non-alcoholic and alcoholic fatty liver diseases. Transl Gastroenterol Hepatol.

[5] Polyzos SA, Kang ES, Boutari C, Rhee EJ, Mantzoros CS (2020). Current and emerging pharmacological options for the treatment of nonalcoholic steatohepatitis. Metabolism.

[6] Jeznach-Steinhagen A, Ostrowska J, Czerwonogrodzka-Senczyna A, Boniecka I, Shahnazaryan U, Kuryłowicz A (2019). Dietary and pharmacological treatment of nonalcoholic fatty liver disease. Medicina.

[7] Abbas G, Haq QMI, Hamaed A, Al-Sibani M, Hussain H (2020). Glucagon and glucagon-like peptide-1 receptors: promising therapeutic targets for an effective management of diabetes mellitus. Curr Pharm Des.

[8] Seghieri M, Christensen AS, Andersen A, Solini A, Knop FK, Vilsbøll T (2018). Future perspectives on GLP-1 receptor agonists and GLP-1/glucagon receptor co-agonists in the treatment of NAFLD. Front Endocrinol.

[9] Sargeant JA, Henson J, King JA, Yates T, Khunti K, Davies MJ (2019). A review of the effects of glucagon-like peptide-1 receptor agonists and sodium-glucose cotransporter 2 inhibitors on lean body mass in humans. Endocrinol Metab.

[10] Mouries J, Brescia P, Silvestri A, Spadoni I, Sorribas M, Wiest R, Mileti E, Galbiati M, Invernizzi P, Adorini L (2019). Microbiota-driven gut vascular barrier disruption is a prerequisite for non-alcoholic steatohepatitis development. J Hepatol.

[11] Nadkarni P, Chepurny OG, Holz GG (2014). Regulation of glucose homeostasis by GLP-1. Prog Mol Biol Transl Sci.

[12] Rowlands J, Heng J, Newsholme P, Carlessi R (2018). Pleiotropic effects of GLP-1 and analogs on cell signaling, metabolism, and function. Front Endocrinol.

[13] Garvey WT, Birkenfeld AL, Dicker D, Mingrone G, Pedersen SD, Satylganova A, Skovgaard D, Sugimoto D, Jensen C, Mosenzon O (2020). Efficacy and safety of liraglutide 3.0 mg in individuals with overweight or obesity and type 2 diabetes treated with Basal insulin: The SCALE insulin randomized controlled trial. Diabetes Care.

[14] Kuchay MS, Krishan S, Mishra SK, Choudhary NS, Singh MK, Wasir JS, Kaur P, Gill HK, Bano T, Farooqui KJ, Mithal A (2020). Effect of dulaglutide on liver fat in patients with type 2 diabetes and NAFLD: randomised controlled trial (D-LIFT trial). Diabetologia.

[15] Budd J, Cusi K (2020). Role of agents for the treatment of diabetes in the management of nonalcoholic fatty liver disease. Curr Diab Rep.

[16] Seo MH, Lee J, Hong SW, Rhee EJ, Park SE, Park CY, Oh KW, Park SW, Lee WY (2016). Exendin-4 inhibits hepatic lipogenesis by increasing β-catenin signaling. PLoS ONE.

[17] Mishra K, Kanduri C (2019). Understanding long noncoding RNA and chromatin interactions: what we know so far. Noncoding RNA.

[18] Sulaiman SA, Muhsin NIA, Jamal R (2019). Regulatory non-coding RNAs network in non-alcoholic fatty liver disease. Front Physiol.

[19] Huang F, Liu H, Lei Z, Li Z, Zhang T, Yang M, Zhou K, Sun C (2020). Long noncoding RNA CCAT1 inhibits miR-613 to promote nonalcoholic fatty liver disease via increasing LXRα transcription. J Cell Physiol.

[20] Yan C, Chen J, Chen N (2016). Long noncoding RNA MALAT1 promotes hepatic steatosis and insulin resistance by increasing nuclear SREBP-1c protein stability. Sci Rep.

[21] Liu J, Tang T, Wang GD, Liu B (2019). LncRNA-H19 promotes hepatic lipogenesis by directly regulating miR-130a/PPARγ axis in non-alcoholic fatty liver disease. Biosci Rep.

[22] Jin SS, Lin XF, Zheng JZ, Wang Q, Guan HQ (2019). lncRNA NEAT1 regulates fibrosis and inflammatory response induced by nonalcoholic fatty liver by regulating miR-506/GLI3. Eur Cytokine Netw.

[23] Alkhatatbeh MJ, Lincz LF, Thorne RF (2016). Low simvastatin concentrations reduce oleic acid-induced steatosis in HepG(2) cells: an in vitro model of non-alcoholic fatty liver disease. Exp Ther Med.

[24] Han J, Liu S, Sun Z, Zhang Y, Zhang F, Zhang C, Shang D, Yang H, Su F, Xu Y (2017). LncRNAs2Pathways: Identifying the pathways influenced by a set of lncRNAs of interest based on a global network propagation method. Sci Rep.

[25] Eißmann M, Gutschner T, Hämmerle M, Günther S, Caudron-Herger M, Groß M, Schirmacher P, Rippe K, Braun T, Zörnig M, Diederichs S (2012). Loss of the abundant nuclear non-coding RNA MALAT1 is compatible with life and development. RNA Biol.

[26] Chen H (2020). Nutrient mTORC1 signaling contributes to hepatic lipid metabolism in the pathogenesis of non-alcoholic fatty liver disease. Liver Res.

[27] Liao S, Yu C, Liu H, Zhang C, Li Y, Zhong X (2019). Long non-coding RNA H19 promotes the proliferation and invasion of lung cancer cells and regulates the expression of E-cadherin, N-cadherin, and vimentin. Onco Targets Ther.

[28] Mundi MS, Velapati S, Patel J, Kellogg TA, Abu Dayyeh BK, Hurt RT (2020). Evolution of NAFLD and Its Management. Nutr Clin Pract.

[29] Montandon SA, Somm E, Loizides-Mangold U, de Vito C, Dibner C, Jornayvaz FR (2019). Multi-technique comparison of atherogenic and MCD NASH models highlights changes in sphingolipid metabolism. Sci Rep.

[30] Katsagoni CN, Papatheodoridis GV, Ioannidou P, Deutsch M, Alexopoulou A, Papadopoulos N, Papageorgiou MV, Fragopoulou E, Kontogianni MD (2018). Improvements in clinical characteristics of patients with non-alcoholic fatty liver disease, after an intervention based on the Mediterranean lifestyle: a randomised controlled clinical trial. Br J Nutr.

[31] Richards P, Parker HE, Adriaenssens AE, Hodgson JM, Cork SC, Trapp S, Gribble FM, Reimann F (2014). Identification and characterization of GLP-1 receptor-expressing cells using a new transgenic mouse model. Diabetes.

[32] Yokomori H, Ando W (2020). Spatial expression of glucagon-like peptide 1 receptor and caveolin-1 in hepatocytes with macrovesicular steatosis in non-alcoholic steatohepatitis. BMJ Open Gastroenterol.

[33] Gupta NA, Mells J, Dunham RM, Grakoui A, Handy J, Saxena NK, Anania FA (2010). Glucagon-like peptide-1 receptor is present on human hepatocytes and has a direct role in decreasing hepatic steatosis in vitro by modulating elements of the insulin signaling pathway. Hepatology.

[34] Kopp F, Mendell JT (2018). Functional classification and experimental dissection of long noncoding RNAs. Cell.

[35] Tian Q, Yan X, Yang L, Liu Z, Yuan Z, Shen Z, Zhang Y (2020). lncRNA NORAD promotes hepatocellular carcinoma progression via regulating miR-144-3p/SEPT2. Am J Transl Res.

[36] Chen H, Xia W, Hou M (2020). LncRNA-NEAT1 from the competing endogenous RNA network promotes cardioprotective efficacy of mesenchymal stem cell-derived exosomes induced by macrophage migration inhibitory factor via the miR-142-3p/FOXO1 signaling pathway. Stem Cell Res Ther.

[37] Wang X (2018). Down-regulation of lncRNA-NEAT1 alleviated the non-alcoholic fatty liver disease via mTOR/S6K1 signaling pathway. J Cell Biochem.

[38] Fu X, Zhu J, Zhang L, Shu J (2019). Long non-coding RNA NEAT1 promotes steatosis via enhancement of estrogen receptor alpha-mediated AQP7 expression in HepG2 cells. Artif Cells Nanomed Biotechnol.

[39] Sookoian S, Flichman D, Garaycoechea ME, San Martino J, Castaño GO, Pirola CJ (2018). Metastasis-associated lung adenocarcinoma transcript 1 as a common molecular driver in the pathogenesis of nonalcoholic steatohepatitis and chronic immune-mediated liver damage. Hepatol Commun.

[40] Bartonicek N, Maag JLV, Dinger ME (2016). Long noncoding RNAs in cancer: mechanisms of action and technological advancements. Mol Cancer.

[41] Chen C, Shu L, Zou W (2019). Role of long non-coding RNA TP73-AS1 in cancer. Biosci Rep.

[42] Ariel I, Miao HQ, Ji XR, Schneider T, Roll D, de Groot N, Hochberg A, Ayesh S (1998). Imprinted H19 oncofetal RNA is a candidate tumour marker for hepatocellular carcinoma. Mol Pathol.

[43] Wang H, Cao Y, Shu L, Zhu Y, Peng Q, Ran L, Wu J, Luo Y, Zuo G, Luo J (2020). Long non-coding RNA (lncRNA) H19 induces hepatic steatosis through activating MLXIPL and mTORC1 networks in hepatocytes. J Cell Mol Med.

[44] Dong CY, Cui J, Li DH, Li Q, Hong XY (2018). HOXA10-AS: A novel oncogenic long non-coding RNA in glioma. Oncol Rep.

[45] Yan X, Cong B, Chen Q, Liu L, Luan X, Du J, Cao M (2020). Silencing lncRNA HOXA10-AS decreases cell proliferation of oral cancer and HOXA10-antisense RNA can serve as a novel prognostic predictor. J Int Med Res.

[46] Tomah S, Alkhouri N, Hamdy O (2020). Nonalcoholic fatty liver disease and type 2 diabetes: where do Diabetologists stand?. Clin Diabetes Endocrinol.

[47] McCommis KS, Finck BN (2019). Treating hepatic steatosis and fibrosis by modulating mitochondrial pyruvate metabolism. Cell Mol Gastroenterol Hepatol.

[48] Fujii H, Kawada N, Japan Study Group of Nafld (2020). The role of insulin resistance and diabetes in nonalcoholic fatty liver disease. Int J Mol Sci.

[49] Santoleri D, Titchenell PM (2019). Resolving the Paradox of Hepatic Insulin Resistance. Cell Mol Gastroenterol Hepatol.

[50] Nair B, Nath LR (2020). Inevitable role of TGF-β1 in progression of nonalcoholic fatty liver disease. J Recept Signal Transduct Res.

[51] Kanda T, Goto T, Hirotsu Y, Masuzaki R, Moriyama M, Omata M (2020). Molecular mechanisms: connections between nonalcoholic fatty liver disease, steatohepatitis and hepatocellular carcinoma. Int J Mol Sci.

[52] Kim S, Park S, Kim B, Kwon J (2016). Toll-like receptor 7 affects the pathogenesis of non-alcoholic fatty liver disease. Sci Rep.

[53] Ye X, Kong W, Zafar MI, Zeng J, Yang R, Chen LL (2020). Plasma vascular endothelial growth factor B is elevated in non-alcoholic fatty liver disease patients and associated with blood pressure and renal dysfunction. EXCLI J.

[54] Pydyn N, Miękus K, Jura J, Kotlinowski J (2020). New therapeutic strategies in nonalcoholic fatty liver disease: a focus on promising drugs for nonalcoholic steatohepatitis. Pharmacol Rep.

[55] Kořínková L, Pražienková V, Černá L, Karnošová A, Železná B, Kuneš J, Maletínská L (2020). Pathophysiology of NAFLD and NASH in experimental models: the role of food intake regulating peptides. Front Endocrinol.

[56] Abdelmagid SA, Clarke SE, Nielsen DE, Badawi A, El-Sohemy A, Mutch DM, Ma DW (2015). Comprehensive profiling of plasma fatty acid concentrations in young healthy Canadian adults. PLoS ONE.

